# Teaching load – a barrier to digitalisation in higher education? A position paper on the framework surrounding higher education medical teaching in the digital age using Bavaria, Germany as an example

**DOI:** 10.3205/zma001180

**Published:** 2018-08-15

**Authors:** Christoph Müller, Saskia Füngerlings, Daniel Tolks, E-Learning working group in the Competence Network Medical Education in Bavaria

**Affiliations:** 1 Julius-Maximilians-University Würzburg, Dean's Office of the Faculty of Medicine, Würzburg, Germany; 2 University Hospital, Ludwig-Maximilians-University Munich, Institute for Didactics and Educational Research in Medicine, Munich, Germany; 3 Leuphana University Lüneburg, Centre for Applied Health Sciences, Lüneburg, Germany

**Keywords:** teaching load, e-learning, digitalisation, blended learning, medical education

## Abstract

This position paper describes the legal framework requirements when crediting digital teaching formats towards the teaching load in higher education medical teaching, as exemplified by the Federal State of Bavaria in Germany. It reveals the need for precise rules adapted to the advances in technology, if the process of digitalisation in higher education (HE) is not to come to a halt.

If HE institutions are to act as centres of innovation with respect to the implementation of digital teaching and learning formats, then structural and strategic positioning with regard to e- and blended learning above all is called for in addition to financial resources, as well as the distribution and sustainable incorporation of digital offerings in faculties and HE institutions. There is a great deal of insecurity however with respect to the legal framework requirements and how best to count digital teaching towards one’s own teaching load. This results to some extent from the complexity of current laws and regulations partially overtaken by didactic and methodological changes in education, with decentralised educational federalism only adding to the complexity.

Bearing in mind teaching and learning formats that are undergoing change or have already been transformed, ways of adapting the (legal) framework to the digital shift need to be found, last but not least in order to offer enthusiastic teaching staff incentives to develop and expand digital formats.

## Zusammenfassung

Dieses Positionspapier beschreibt die rechtlichen Rahmenbedingungen bei der Anrechnung digitaler Lehrformate auf das Lehrdeputat in der medizinischen Hochschulbildung am Beispiel Bayern. Es zeigt die Notwendigkeit präziser, dem technologischen Fortschritt angepasster Regelungen, wenn der Digitalisierungsprozess in der Hochschullehre nicht ins Stocken geraten soll.

Wenn Hochschulen als Orte der Innovation im Einsatz digitaler Lehr- und Lernformate fungieren sollen, bedarf es neben finanzieller Ressourcen vor allem einer strukturellen und strategischen Positionierung im Bereich des E- und Blended-Learnings sowie der Verbreitung und nachhaltigen Verankerung digitaler Angebote innerhalb der Fakultäten und Hochschulen. Hinsichtlich der rechtlichen Rahmenbedingungen und des Umgangs mit digitaler Lehre bei der Anrechnung auf das eigene Lehrdeputat herrscht jedoch große Unsicherheit, was unter anderem an der komplexen, vom didaktisch-methodischen Wandel der Lehre in Teilen überholten Gesetzes- und Verordnungslage liegt, die durch den dezentralen Bildungsföderalismus an Komplexität dazugewinnt.

Unter Berücksichtigung veränderter und im Wandel begriffener Lehr- und Lernformate sollen Wege gefunden werden, die (rechtlichen) Rahmenbedingungen dem digitalen Wandel anzupassen, um nicht zuletzt engagierten Lehrenden Anreize zum Ausbau digitaler Formate zu bieten.

## Introduction

Digitalisation has a wide-ranging influence on our everyday lives and has triggered a transformation in nearly every aspect of society [1], [2]. This shift also leads to fundamental changes in the organisational structures of teaching and learning, as well as the roles and requirements profiles of students, teachers, and employees of higher education institutions. In the process, new didactic opportunities and room for manoeuvre with respect to the dissemination of competence and knowledge emerge, but also challenges to which higher education institutions need to react correspondingly [3], [4]. According to the German Forum for Higher Education in the Digital Age, the structural conditions at higher education institutions are in principle not unfavourable to the development of innovations in the field of digital media, given for instance the high degree of autonomy through decentralised institutes and chairs that can promote innovation [4]. It should not go without stating that higher education institution management needs to share the responsibility of digitalisation, if comprehensive digitalisation processes that reach deeply into the competence of both teachers and students are to be implemented successfully. In this manner, the application of digital teaching and learning methods is establishing itself slowly in higher education and yet remains distributed in a very heterogenic fashion among institutions [2]. If higher education establishments are to act as places of innovation with regard to the application of digital media in teaching, not only the availability of financial resources but also the structural and strategic distribution and embedment of digital teaching and learning activities within institutions are of great importance [4]. According to the final report published by the German Forum for Higher Education in the Digital Age, higher education policy has recognised that the general framework for higher education must be developed further. However, great uncertainty prevails with respect to the legal framework associated with digital teaching in particular [2]. On the one hand, this is due to decentralised educational federalism, which leads to discrepancies in the manner in which digital higher education is recognised as a result of differences between federal state higher education laws and teaching regulations as well as variations in the regulations of individual establishments [4]. On the other hand, the ability to credit digital teaching and learning towards one’s teaching load, the handling of digital examinations, or questions of copyright with respect to the creation and use of teaching and learning materials for that matter are often fundamentally regulated and conveyed insufficiently [3], [4]. This is one of the reasons why digital teaching and learning are not promoted at many German institutions of higher education [4].

Within the framework of the European University Association (EUA) Conference, representatives of higher education establishments in Europe have consequently ascertained and criticised that digitalisation is being impeded both on a national and international level, with complex support regulations, data protection regulations that are out of date, as well as rules demanding the physical presence of teaching staff being cited. The Conference participants call for closer inter-institutional collaboration, both nationally and internationally [5]. According to the Horizon Report in 2014, studies running in parallel to the programme in Europe have also revealed that inflexible, state-run structures and budgets, as well as the lack of incentives for innovative teaching staff, are factors hindering the dissemination of new methods of learning [6]. As a result, Arnold and colleagues demand that the teaching load be newly structured, in order to take into account the considerable and varying time-related pressures, resulting from the preparation of content appropriate to the media employed, the co-operation necessary during the production of the media, as well as the asynchronous communication with students [7]. Bischof and von Stuckrad talk of a “sleeping revolution”, inhibited by a plethora of institutional barriers [1]. They demand that policy makers abolish such barriers systematically and align the legal framework with technological advancement. Likewise, higher education institutions are to adopt digitalisation as a strategic task, which, if nothing else, results from their self-conception as an institution and their social mandate [1], [2].

In this paper, we view the legal and structural framework when deducing a teacher’s workload on implementation of digital teaching formats, exemplified by the Regulation of Teaching Duties of the Federal State of Bavaria, Germany, which is referred to as representative and exemplary for the total number of 16 regulations of teaching duties valid in the Federal Republic of Germany. We thus wish to make a contribution towards clarifying the insecurity surrounding the deduction of a teacher’s workload and remove a barrier to the implementation of novel teaching concepts. In total, this should lead to the consideration of digital teaching formats acting as a driving factor in the digitalisation of teaching in higher education when deducing the teaching load in future. Furthermore, we recommend that standardisation be introduced to deal with the current differences when handling the recognition of digital higher education.

Finally, we would like to point out that the common, traditional teaching forms are by no means to be replaced as a result of increases in the appreciation of e-learning activities in education following improved flexibility in terms of the legal framework, focussing instead on changing the culture of teaching and learning with the aim of improving the quality of degree courses and teaching.

## 1. Definition and current status of e-learning

Bearing in mind the common definitions, e-learning may be defined for the following statements as a “generic term encompassing all the means in which digital media are employed for the purposes of teaching and learning, be it via some digital storage medium or the Internet, perhaps to impart knowledge, to exchange between people, or to work together on digital fragments” [8]. Characteristics essential to electronic learning are asynchronous and synchronous communication options, multi- and hypermediality of teaching and learning content, as well as the provision of materials in virtual classrooms that are available to teachers and students alike independent of time and location [7].

For contemporary and future students who have grown up or are growing up with technological devices, “Learning and mediality are inextricably linked with one another” [9]. In conjunction with the ubiquitous demand for stimulating and student-centred teaching, notably attainable through the implementation of digital media and adaptable to the realities of life of an increasingly heterogeneous student population, higher education establishments rate particularly highly as “places of innovation in the implementation of digital media in education” [4]. According to the objectives laid down in the strategy for a digital future compiled by the Bavarian State Government, institutions of higher education are to “become “digital campuses”, on which all the protagonists of science are to use the opportunities and possibilities arising from digitalisation and profit from them” [10].

## 2. E-Learning in medical education

Novel digital scenarios, concepts, and methods are increasingly finding their way into teaching in medical education [11]. Growing importance is being ascribed in healthcare to training and the acquisition of both profession-specific as well as interprofessional competencies, for which intrinsic teaching formats need to be tested and developed [12]. In addition to lectures, the Regulation governing the acquisition of the licence to practice medicine that is mandatory in the teaching of medical students (ÄApprO) explicitly mentions seminars as well as practical exercises, including bedside teaching, practical training, and clinical rotations, as preferred forms of course (§2 para. 1 ÄApprO) [13]. Concrete methods or forms of teaching are not mentioned. Consequently, the term “e-learning” is neither to be found in this form nor in synonyms or paraphrases. The great number of successful (above all commercial) offerings of e-learning is a clear indicator of how meaningful it is to implement digital media within the framework of medical teaching and vocational training, as well as its relevance to practical, hands-on training. Against this backdrop, it may at first be surprising that the proportion of electronic teaching and learning content in medical education at state-funded institutions of higher education is relatively low and is implemented to differing degrees at faculties of medicine [14], [15]. However, on closer inspection, it is clear that the “financing of digitalisation initiatives” in medicine, as in other fields of science, exhibits a “very large proportion of external project financing”, but “financial incentive structures aiming to anchor digital teaching and learning formats in higher education sustainably and in a structurally strategic fashion” are often lacking or limited by unattractive framework conditions [4]. Furthermore, the dualism of research and teaching on the one side and patient care on the other side characteristic of university-based medicine [16] leads to a “triple burden” on physicians working in research and also engaged in teaching activities. Educational trends will also increasingly find their way into medical degree courses: the growing significance of self-study, group and project work (shift from teaching to learning), the setting of competence-oriented examinations in conjunction with one’s studies ((e)-assessment), the integration of the virtual component into the learning environment (virtual learning environment), and the development of the campus into a place of learning (mobile learning) [17]. This leads to a change in the role of the teacher, who is “no more the single provider of knowledge and information,” as “these items are now sought, developed, and discussed collectively” [18]. This paradigm shift of teachers from knowledge mediators to “knowledge providers”, advisors, and moderators may be supported meaningfully and decisively by the implementation of digital teaching formats. According to Rummler, the methods, learning pathways, and strategies of students are being transformed by media, which results in the need to readjust the pedagogic interventions [19]. Despite the relatively gloomy inventory, the most recent developments at institutions of higher education in medicine indicate at least that new and innovative technology-based learning and teaching methods such as the inverted-classroom method are slowly establishing themselves [20].

## 3. Provisions regarding capacity and public sector employment law with respect to crediting e-learning towards teaching load in accordance with the Regulation of Teaching Duties of the Federal State of Bavaria, Germany (LUFV)

### 3.1. The tension between federalism and idealism

As previously stated, German education federalism leads to “immense differences in both state higher education laws and teaching regulations as well as between individual establishments of higher education with respect to possible rules governing the promotion and recognition of digital higher education” [4]. This lack of uniformity and partial lack of transparency in the legal framework unnerves teaching staff and allows the digitalisation of teaching to stagnate [21].

The experience of higher education employees in the e-learning sector coincides with the studies undertaken by Bernd Kleimann, according to which the “time required to develop, implement, and maintain e-learning” [22] may be found through three means:

- through “voluntary additional work” by lecturers, who enrich their materials with electronic teaching and learning units motivated by idealism,
- through the “reduction of research and self-administration activities,” by which a process of restructuring takes place and one’s involvement in other areas is reduced as a result of prioritisation,
- or through the “reduction of face-to-face teaching,” in which e-learning activities count towards teaching load and where applicable replace face-to-face teaching [22].

Whereas the first two named scenarios have negative consequences in the medium and long term – “in case a) with respect to teachers’ work satisfaction, in case b) with respect to the achievements in research and self-administration” – there is broad consensus that it is necessary to compensate for the additional work resulting from the digitalisation of education, which should be realised through the ability to credit towards teaching load. In addition, it is possible, “through a manageable amount of additional work by teaching staff in media-based education, to realise a time-saving effect that overcompensates the additional work required” [22].

#### 3.2. Provisions of the Regulation of Teaching Duties of the Federal State of Bavaria, Germany (LUFV)

The regulation of teaching duties for scientific and artistic personnel at universities, art colleges, and universities of applied sciences (Regulation of Teaching Duties – LUFV) dated 14 February, 2007 (GVBl S.201, BayRS 2030-2-21-WFK) in the currently valid version and legally binding for all teaching staff at state universities and university hospitals, stipulates the requirements of public sector personnel law when determining teaching load [23]. Leaving out some detailed (special) rules, the following provisions are applicable to medical education at universities and university hospitals: The duration of a teaching session has to be at least 45 minutes of teaching per week within the teaching period of the semester (§2 para. 1). Teaching sessions that are not expressed as weekly sessions per semester (SWS) or not spread across every week of the semester teaching period are to be converted into SWS; in such cases, the sum of the individual sessions is to be divided by the number of weeks the semester teaching period is in duration (§3 para. 6). Furthermore, it is important to note that the extent of the teaching load needs to be calculated in such a way as to ensure that “not only keeping the teaching material up to date, but also the modernisation of the means of communication is kept within the time periods for preparation and follow-up,” which is why the “provision of informative and teaching material in accompaniment with face-to-face teaching will not have any effect on the extent of the teaching load” [24].

With regard to specific weighting factors, one differentiates between lectures, seminars, and exercises, as well as “their modern, particularly Internet-based form” that are weighted fully (= factor 1.0) towards the teaching load, colloquia and revision courses by a factor of 0.7, and field trips by a factor of 0.3. “Other courses” – not further specified in the following – are weighted by a factor of 0.5 when counted towards the teaching load (§3 para. 2) [23]. Practical training, common to medicine – declared as a “practical exercise” in the ÄApprO (ÄApprO §2 para. 1), which is not named in the LUFV, is viewed in the following as analogue to lectures, exercises, and seminars and correspondingly weighted by a factor of 1.0. 

The possibility of having “modern” and “Internet-based” forms of lectures, exercises, and seminars may be valued as fundamental approval of the fact that these named types of course can be held virtually. The decision as to whether teaching may be provided digitally is “initially a question of the methodological composition, upon which the teachers themselves decide alone,” according to the Conference of the State Ministers for Culture and Education in Germany [24].

Moreover: if students do not require constant supervision, the course is counted towards teaching load weighted with a factor of 0.3 (ibid.). Courses in which two or more teachers are involved are only counted once in total – each individual teacher involved in teaching the course being credited in correspondence with the proportion of their respective involvement. Insofar as a course is taught by multiple disciplines, it may be counted a maximum of twice in total for the participating teaching staff (§3 para. 7) (see Table 1 (Tab. 1)).

In order to calculate the semester periods per week (SWS) of individual types of course, the following formulae are available, which are applied to the declaration of actual teaching load achieved during each semester:

Formula to calculate weekly courses with equal numbers of sessions: 


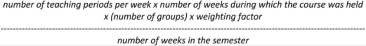


Formula to calculate courses taking place at irregular intervals, block courses, or field trips: 


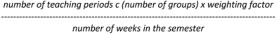


Generally speaking, the teaching load may be fulfilled partially by the implementation and supervision of multimedia-based courses [24] that are considered as “supplementary offers” and not constituents of the list in Table 4 (Tab. 2) of teaching course types stipulated by the curriculum. 

In such cases, the following provisions apply: “The creation and supervision of multimedia-based courses may be counted towards the teaching load to an extent respective of the time involved, but only to a maximum of 25 percent of the determined teaching load. One teaching period or session (weighting factor of 1) corresponds to 3 working hours” (§3 para. 9).

In specific terms and as a consequence, a maximum of one quarter of the total teaching load in hours may be spent on the creation and supervision of a multimedia project, whereby – in analogy to courses taking place irregularly or block courses – the calculation is performed in terms of working hours completed. Sample calculation: A teacher with a teaching load of 10 SWS would like to develop a multimedia-based course. According to §3 para. 9, he may spend a maximum of 25% of his teaching load on this project, in his case 2.5 SWS. The effort required to develop and supervise a teaching session can equate to up to three working hours, in which case 7.5 hours per week are available for the multimedia project in this example. Converting this to a working week comprising 40 working hours, the teacher can occupy himself for around one working day per week during the lecturing period of the semester. This may appear as very generous, but nigh on impossible to realise in medical education, as a result of the above-mentioned triple burden on teachers without the corresponding “backing” of one’s supervisor or exemption from duties in other areas of activity.

#### 3.3. Proposition of a course typology taking e-learning formats into account

Given the complete lack of any concrete course typology within the LUFV in the field of electronically assisted teaching, we would like to propose the following typology of teaching and learning forms, allowing for the current legal requirements in force. This typology considers the decisive aspect of supervision, the significant differentiating characteristic in the field of e-learning, and attempts to integrate forms of digital teaching into the current array, in order to provide some initial orientation (see Table 4 (Tab. 2)):

In this connection, it needs stressing that the (often one-off) effort required to create or update e-learning or online materials for the course forms labelled 1-3 cannot be credited. The weighting factor according to the LUFV corresponds exclusively to the actual course duration, although the respective divergence in preparation and follow-up times has already been taken into account by the different factors [24].

The current weighting factors are listed in the typology intentionally, since it is falls into the jurisdiction of legislators to align or amend the weighting factors. In any case, it is desirable to clarify the named course types more precisely, as these remain vague, as in the case of “other courses.” Furthermore, it is highly important to consider hybrid formats in particular such as the inverted-classroom model, since the boundaries between permanently supervised sessions (face-to-face or presence phase) and partially supervised or, where applicable, unsupervised sessions (online phase) that require a large amount of effort to prepare in spite of everything are not fixed.

## 4. Criticism and recommended actions

In view of the digitalisation of society and the relevance of digital media to how students of today study and learn, the structural and strategic conflict in higher education institutions with the political, institutional, and university-wide framework requirements of digital education joins up with the technical, didactic, and methodological question of how to deal with digital media in education. According to Jäckel, the expectations of both teachers and students placed on technical equipment are growing faster than they can be fulfilled, which leads to the fact that such infrastructure is involuntarily experienced as being deficient [25]. In this regard, the German Forum for Higher Education in the Digital Age is calling for “continued substantial efforts on all levels, to exploit the benefits of digitalisation at German institutions of higher education all across the country” [2]. Against this backdrop, it is no wonder that although clarification of the separate regulations of teaching duties and ideally the unification of these regulations across Germany’s state borders represent an important step towards greater transparency with regard to the legal framework, they fail to address the problem on their own. Further measures directed at different protagonists in higher education establishments are needed in addition, to drive the digitalisation of teaching forward sustainably.

### 4.1. Legal provisions

As stated at the outset, there is a huge divide between the political intentions to promote and drive forward digitalisation in higher education and the status quo of digital education that exists in faculties. Moreover, the current provisions in Germany are exceptionally heterogeneous. To add to the difficulties, the legal position for the group of scientists that wishes to implement digital teaching and learning is also little known. Furthermore, if one takes up the fact that the time scientific staff members are employed at a higher education institution is comparatively short and that scientists change their university employers in many cases – also across Federal state borders – for the purpose of professional development, then the unification of the 16 sets of regulations governing teaching duties is desirable. With current funding projects as a backdrop, for example the “Federal and States programme Quality Pact in Education (QPL)” from the German Federal Ministry of Education and Research (BMBF) that is financing a total of 71 universities, 61 universities of applied science, as well as 24 art colleges and universities of music with a variety of individual projects also in the field of e- and blended learning during the current period until 2020, the existence of 16 regulations governing teaching duties does appear rather old-fashioned.

The current provisions are to be judged as inadequate and particularly inhibit the innovative capacity of new teaching and learning scenarios. For example, purely online-based courses such as a MOOC (massive open online course) would not be counted fully towards teaching load in this fashion. The offsetting of the teaching load for novel and very progressive methods of teaching and learning such as the development and implementation of serious games [26] or virtual reality applications [27] cannot currently be portrayed. Blended learning courses following the flipped- or inverted-classroom approach lie in a grey zone, since the legal provisions regarding the creditability of the individual phase in which the content to be transferred is made available in a digitally processed form are difficult to interpret. Consequentially, only those members of the scientific staff with greater-than-average access to resources and/or high level of motivation, and who are prepared now and then to spend time on the development of digital teaching also outside of their regular working hours will be able to launch new, innovative projects, as is mentioned in the article by Oliver Janoschka [28]. 

The current provisions conceal the dangers that motivated employees who make a particular effort to implement new media into higher education may not fulfil their teaching load on the one hand, and in the worst case on the other hand make themselves redundant, as the initially time-intensive effort required to create new content can ultimately be overcompensated for in the following semesters and have a time-saving effect [22]. 

Any legal reorganisation needs to be carried out with caution as well as include and reflect adequately the new framework conditions for a transformation in studying behaviour. This is precisely because the creditability of multimedia production and online teaching towards the teaching load is viewed by teachers as an effective measure to increase the use of e-learning, at least in the opinion of 78% of 201 higher education establishments approached within the framework of the “E-Readiness” Survey carried out by HIS GmbH and the Multimedia Kontor Hamburg [21]. Only in this way can higher education institutions once again fulfil their actual intended role as driver of innovation.

Digital teaching, as exemplified by online phases within the framework of inverted-classroom (IC) courses, should be viewed on an equal footing with traditional course forms and correspondingly counted towards the teaching load: If a seminar is held in accordance with the IC model, then the times of attendance of, for example, 2 SWS remain unchanged; however, the conception, creation, and supervision of the online phase represents a significant increase in the workload for teachers. The workload is also increased for students, as the online phases are essential to the preparation of the periods of actual attendance and require an appropriate amount of time to prepare and process. It is only logical that IC courses will need to be furnished with a greater number of SWS than conventional seminars. However, the creation and supervision of a multimedia project in accordance with §3 para. 9 of the LUFV should count towards one’s own teaching load to a greater extent than 25% in justified cases, for example when a large degree of creative effort is necessary, or the digital subject matter is complex in content or technically very demanding. In this context, it is sensible to differentiate between the actual time-intensive creation of a new digital project and the supervision of a current project. Thus, a compromise could well include fundamentally increasing the hours that count towards the teaching load to 50% when a multimedia project is created, while the applicable upper limit for supervision of a project remains at 25% or perhaps 50% likewise in justifiable cases.

#### 4.2. Higher education institutions and faculties

Studies reveal that “the fundamental organisational infrastructure for digital teaching is available at a large number of higher education institutions in Germany,” but that this “can differ substantially depending on the type of institution, its sponsorship, and its size,” [29]. This is in no way surprising, given the educational federalism and the largely heterogenic handling of digital teaching at higher education establishments and faculties.

The legal provisions, which have been mentioned several times and in part criticised for not having been adapted adequately to the advancement of technology, allow higher education on the other hand great room for manoeuvre when dealing with digital teaching, precisely because of their ambiguity and lack of definition. At this point, we therefore advocate generosity when interpreting the regulation of teaching duties, in order to support innovative projects and protagonists of e-learning, and reward the input of committed teachers.

Furthermore, the recommendation is to compose a directive, which is binding for the entire university or at least faculty and that contains aspects relating to capacity, public-sector employment law, and copyright with respect to digital teaching. This may serve teaching staff as a definitive guide among other things with regard to the creditability of digital teaching formats by also ideally including tangible and contemporary good-practice examples that are not portrayed by the legal provisions. The strategy paper, “Crediting E-Learning at the UDE,” from the University of Duisburg-Essen is a good example of this that explains and comments on the legal requirements of the Regulation of Teaching Duties of the State of North Rhine-Westphalia, as well as defining an e-learning strategy for the entire University [30]. 

The competitiveness and attractiveness of a higher education institution is inextricably linked to its prestige or reputation and “numerous establishments of higher education anticipate an increase in reputation through the use of e-learning” [21].

In this context, the “adherence to relevant agreements on objectives at faculty level”, “minimum proportions of e-learning courses in the curriculum”, “internal benchmarks between faculties”, “media reporting of exemplary e-teaching on offer”, or “excellent evaluation results” are named as suitable measures (ibid.), which have an effect on the attractiveness of digital teaching to teaching staff, be it through awards and teaching prizes, certification marks, e-learning certificates, or e-teaching awards.

#### 4.3. Co-operation projects

Co-operation projects, for example the joint development, implementation, and supervision of digital teaching and learning formats on the platform of the Virtual University of Bavaria (vhb) or within the framework of other networks, are beneficial in two ways in this context: firstly, a co-operation project leads to scientific exchange between researchers and higher education institutions, but also to distribution of the workload and thus the predictable and quick realisation of particularly extensive digital projects. Secondly, structures of financial incentives make it possible to draw on resources and additional personnel (e.g. in the form of student assistants) for the project.

## 5. Outlook and summary

E-learning or rather digital teaching formats need to adapt to the inevitable advancement of technology and are therefore naturally in a constant state of flux. Although digital teaching is totally indispensable in our society currently, with particular importance being attached to the subject and related topics from the point of view of universities and above all students (29), a lot of room for both discussion and action will also be required by all protagonists in higher education in the future.

If we view only the legal and structural framework, which was the aim of this analysis, we have to note that many questions remain unanswered: what is the impact of online or blended learning courses on students’ study load? Will the SWS count if the teacher is only available online (for example, in a chatroom) and not physically present? Who ensures and how will it be ensured that students actually spent their time online with the digital content? The list of unanswered questions or at least questions that can only be answered imprecisely could be extended, which should be avoided in favour of a list of theses stimulating a suitable approach to digital teaching, which catalogues the above-named criticisms and demands, while further accommodating additional demands.

### Politics

- Raise the percentage creditability of digital teaching formats towards the teaching load.
- More flexible creditability models, in order to represent the constantly advancing development of new teaching and learning technologies and concepts appropriately.
- Harmonisation or standardisation of the provisions for all Federal States in Germany with respect to fluctuations in academic mid-level faculty members.
- Funding and support of new learning and teaching technologies and concepts as well as the sustainable incorporation of digital teaching formats in the curriculum.

#### Higher education institutions and faculties

- Flexible interpretation of the current regulations, in order to support new, innovative projects and create incentives for teachers.
- The development of a transparent e-learning strategy that is applicable ideally throughout the faculty or whole institution.

#### Protagonists

- Enter into co-operations and search for funding and support opportunities, in order to distribute the workload across a number of persons and avoid teaching load restrictions.
- Improved networking (faculty internal and interfaculty as well as university internal and interuniversity) of e-learning protagonists for the purpose of exchanging experience with digital teaching formats and their respective creditability towards teaching load.

The intention of this article is to name the current uncertainties with respect to the creditability of digital teaching formats towards teaching load, enlighten, and contribute towards breaking down an important barrier to the implementation of new teaching concepts. The priority is to demand at least a clarification of the current regulations of teaching duties. That said, the harmonisation of the 16 current sets of regulations in Germany is desperately needed in times of BMBF-funded projects aiming to improve the conditions of study and quality of teaching with foci on the expansion and incorporation of digital teaching formats in light of the personnel fluctuation in mid-level academic faculty in German institutions of higher education.

It would be desirable if calculation of the teaching load were to act as a motivating factor in the digitalisation of higher education in the future and not represent a limiting factor through imprecise regulations and the associated uncertainties on the part of teachers, as is presently the case.

## Competing interests

The authors declare that they have no competing interests. 

## Figures and Tables

**Table 1:**
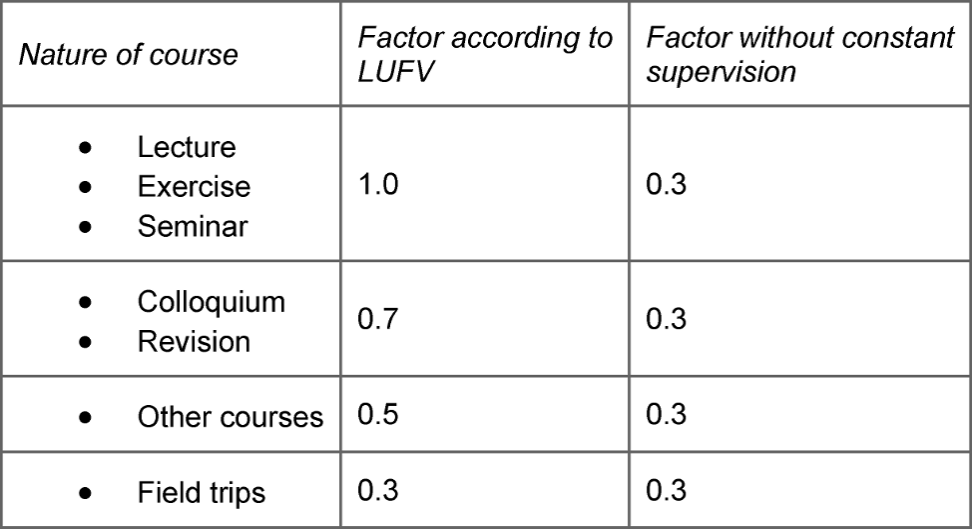
Courses and weighting factors (in accordance with §3 LUFV)

**Table 2:**
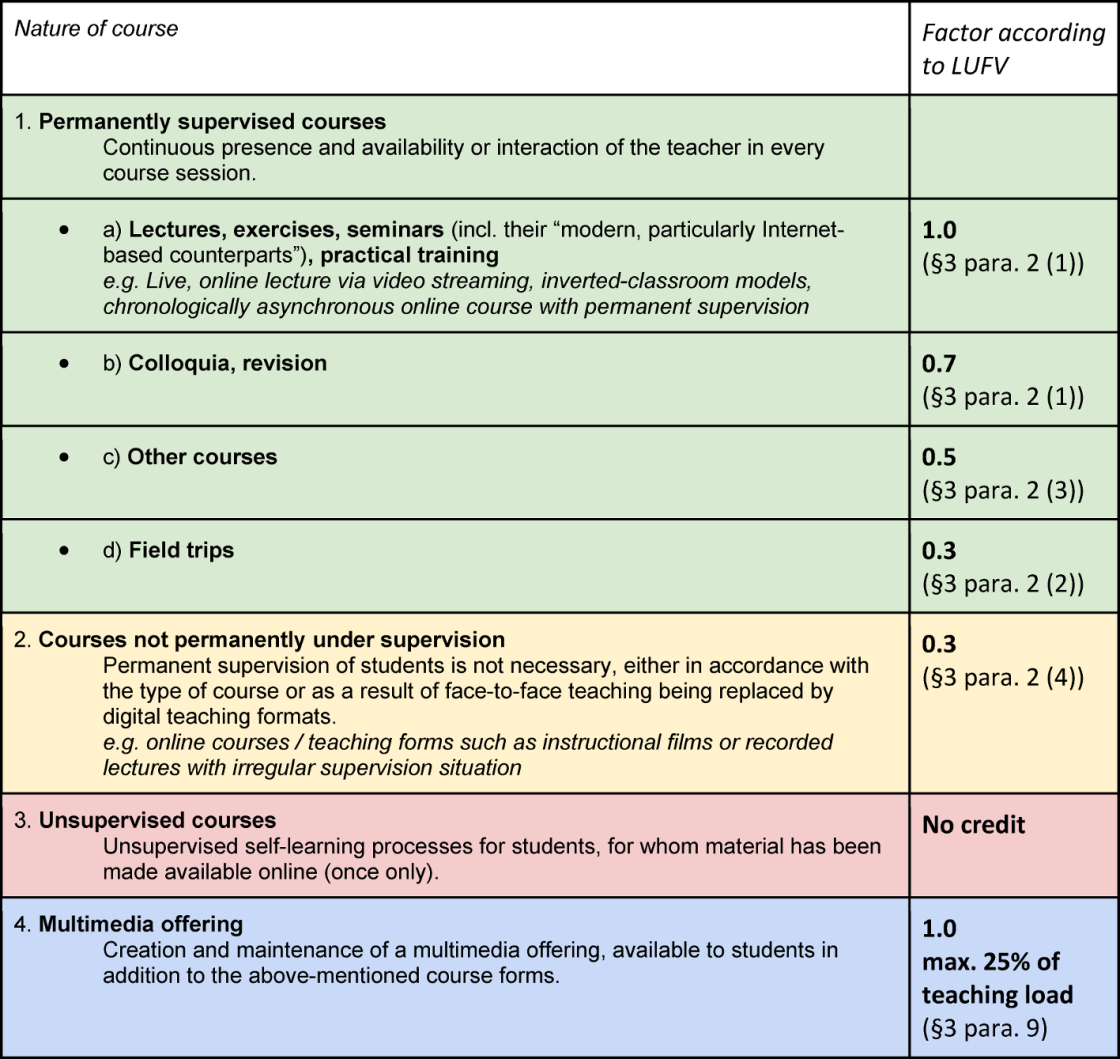
Table 4: Proposed course topology
